# Neonatal Circumcision: What Are the Factors Affecting Parental Decision?

**DOI:** 10.7759/cureus.19415

**Published:** 2021-11-09

**Authors:** Christian G Guevara, Justin K Achua, Ruben Blachman-Braun, Isabella Cabrera-Valencia, George A Ransford, Rafael Gosalbez, Andrew S Labbie, Miguel A Castellan, Alireza Alam

**Affiliations:** 1 Pediatric Urology, Nicklaus Children's Hospital, Miami, USA; 2 Urology, University of Miami Miller School of Medicine, Miami, USA; 3 Urology, Jackson Memorial Hospital, University of Miami Miller School of Medicine, Miami, USA

**Keywords:** circumcision, decision-making process, guardians, children, aap guidelines

## Abstract

Introduction: The American Academy of Pediatrics (AAP) guidelines state that the health benefits of circumcision outweigh the risks, but these benefits are not enough to recommend universal newborn circumcision. Therefore, it is the guardians’ decision to circumcise their son. In this study, we assess the factors that influence the decision-making process for newborn circumcision.

Methods: A prospective study was done from January to April 2020 for newborn circumcision. AAP guidelines were used as an educational tool and given to the parents on the day of patient circumcision assessment. On procedure day, a self-reported survey regarding the reasons for circumcision and the usefulness of the guideline as an educational resource was given to guardians.

Results: A total of 265 parents completed the survey. Of the study variables, the future health of the child and the circumcision status of the father were considered extremely important factors influencing the decision-making process for 168 (63.4%) and 90 (34%) guardians, respectively. The study showed that 226 (85.3%) of the parents found the AAP guidelines helpful whereas 39 (14.7%) did not.

Conclusion: Overall results suggest that the health of the child and the father of the child being circumcised are the primary factors that influence the guardians’ decision to circumcise their child. In addition, providing parents with an educational resource such as the AAP guidelines policy statement prior to circumcision may serve as a way to supplement the discussion between parents and providers.

## Introduction

Male circumcision is the removal of the prepuce [1]. It is one of the most common procedures performed in the world and makes up over 10% of pediatric urology cases [2]. An estimated 58.3% of male newborns and 80.5% of males aged 14-59 years in the United States are circumcised [3,4]. In 2012, the American Academy of Pediatrics (AAP) Task Force on Circumcision, endorsed by the American College of Obstetricians and Gynecologists, released their collective position on circumcision [5,6]. The AAP found that preventative health benefits of elective circumcision of male newborns outweigh the risk of the procedure. Male newborns who undergo circumcision benefit from significant reductions in the risk of urinary tract infections in the first year of life and penile cancer and risk of transmission of HIV and other sexually transmitted infections later in life. The task force found that the benefits of circumcision were enough to justify access to all families and warrant third-party payment [1,5,6]. However, the AAP found that the health benefits were not great enough to recommend routine circumcision for all male newborns and that circumcision remains at the discretion of the guardian. Lastly, they state it is the physician’s responsibility to counsel guardians on the health benefits and risk of newborn circumcision in a fair and unbiased manner [5,6].

Despite the aforementioned benefits, newborn circumcision rates in the United States have declined significantly over the past few decades. Guardians ultimately face the dilemma of deciding whether circumcision is in the best interest of their son. This dilemma extends beyond simply the health of the newborn, encompassing religious, ethical, and cultural beliefs and practices. The objective of this study is to determine the factors that influence the guardians' decision-making process for infant circumcision. We hypothesize that parents usually decide to circumcise their child if the father is also circumcised.

## Materials and methods

After obtaining Nicklaus Children's Hospital IRB's consent, a prospective study among parents seeking an outpatient male newborn circumcision was conducted at our institution from January 2020 to April 2020. During the first newborn circumcision clinic appointment at our pediatric urology offices, shortly after the parents were educated by the provider on the risks and benefits of newborn circumcision, the AAP guidelines policy statement was given to the patient’s guardians. The study was initiated by the provider and parents were consented to participate in the study prior to the end of their appointment. The parents were then instructed to review the AAP guidelines in their entirety prior to the procedure appointment. On procedure day, and an average of one week after the first appointment, a self-reported survey (one per child) regarding the reason for circumcision was handed to parents. In this survey, the usefulness of AAP guidelines as an educational resource was also assessed.

The self-reported survey was administered to all parents who sought circumcision of their newborn son at our institution. Surveys were provided in paper form only and participation was strictly voluntary. The survey contained eight questions: questions one through three assessed the guardians’ demographics, questions four through six focused on assessing the usefulness of the AAP guidelines as an educational resource, and the last two questions assessed the reasons for newborn circumcision. Surveys were completed by the patient’s guardians. Data were collected and managed using Research Electronic Data Capture (REDCap) tools hosted at our institution. Statistical analysis was performed with SPSS, version 24 (IBM SPSS Statistics, Armonk, NY). Categorical variables between groups were presented as absolute values and frequencies, comparisons between groups were performed using the chi-square test. Significance was defined as p-value <0.05.

It is important to note that the study conducted from January 2020 to April 2020 involved the distribution of the American Academy of Pediatrics (AAP) guidelines as part of the educational process for parents seeking newborn circumcision. However, it should be highlighted that the AAP guidelines available during this study were already expired at the time of its execution. Patients participating in the study were promptly informed that the guidelines were no longer current at the time of the study's execution. To facilitate access to the most up-to-date and relevant information, patients were directed to visit the official AAP website. Here, they could readily find the latest guidelines, recommendations, and comprehensive information, thereby enabling them to make informed decisions based on the most current data available.

## Results

A total of 265 guardians completed the survey. The most frequent child race/ethnicity was white (n = 125, 47.2%). Our study showed that the majority of the children had a father (n = 182, 68.7%) and brother (n = 89, 78.1%) who were also circumcised (Table 1). Of the study variables, the future health of the child and the circumcision status of the father were influential factors in the decision-making process for 168 (63.4%) and 90 (34%) guardians, respectively (Table 2). A total of 226 (85.3%) of the parents found the AAP guidelines helpful whereas 39 (14.7%) did not find the guidelines helpful. When comparing the demographic data of those that found the AAP data helpful, it was noticed that younger mothers tend to find the AAP guidelines significantly more useful (p = 0.047). In addition, there was no difference regarding race/ethnicity, father age, history of circumcised father or brother (p < 0.05) between groups that found the AAP guidelines helpful and those that did not.

**Table 1:** Comparison between the parents that considered the AAP guidelines helpful and not helpful. AAP, American Academy of Pediatrics.

	Overall, n = 265 (100%)	Helpful, n = 226 (85.3%)	Not helpful, n = 39 (14.7%)	p-value
Race/ethnicity of the child				
White	125 (47.2%)	105 (46.5%)	20 (51.3%)	
African-American	66 (24.9%)	60 (26.5%)	6 (15.4%)	
American Indian	1 (0.4%)	1 (0.4%)	0	
Asian	7 (2.6%)	6 (2.7%)	1 (2.6%)	
Multiple or other ethnicity	66 (24.9%)	54 (23.9%)	12 (30.8%)	0.621
Age of mother				
<18 years	2 (0.8%)	2 (0.9%)	0	
18-24 years	36 (13.6%)	35 (15.5%)	1 (2.6%)	
25-34 years	145 (54.7%)	125 (55.3%)	20 (51.3%)	
35-44 years	82 (30.9%)	64 (28.3%)	18 (46.2%)	0.047
Age of father				
<18 years	2 (0.8%)	0	2 (0.9%)	
18-24 years	20 (7.5%)	19 (8.4%)	1 (2.6%)	
25-34 years	116 (43.8%)	102 (45.1%)	14 (35.9%)	
35-44 years	110 (41.5%)	90 (39.8%)	20 (51.3%)	
45-54 years	13 (4.9%)	9 (4%)	4 (10.3%)	
55-64 years	1 (0.4%)	1 (0.4%)	0	
>65 years	3 (1.1%)	3 (1.3%)	0	0.329
Father circumcised				
No	83 (31.3%)	74 (32.7%)	9 (23.1%)	
Yes	182 (68.7%)	152 (67.3%)	30 (76.9%)	0.229
Brother circumcised (n = 114)				
No	25 (21.9%)	21 (21.9%)	4 (22.2%)	
Yes	89 (78.1%)	75 (78.1%)	14 (77.8%)	0.974

**Table 2:** Factors that influence the guardians' decision-making process for circumcision.

	Health	Father of the child circumcised	Religion	Cosmetic	Other people I know are circumcised
Prefer not to answer	25 (9.4%)	46 (17.4%)	60 (22.6%)	70 (26.4%)	64 (24.2%)
Not important	3 (1.1%)	51 (19.2%)	116 (43.8%)	71 (26.8%)	76 (28.7%)
Not so important	3 (1.1%)	13 (4.9%)	30 (11.3%)	15 (5.7%)	26 (9.8%)
Somewhat important	9 (3.4%)	30 (11.3%)	23 (8.7%)	49 (18.5%)	32 (12.1%)
Very important	57 (21.5%)	35 (13.2%)	12 (4.5%)	29 (10.9%)	29 (10.9%)
Extremely important	168 (63.4%)	90 (34%)	24 (9.1%)	31 (11.7%)	38 (14.3%)

## Discussion

Many other studies have investigated the factors that influence a guardian’s decision for circumcision, and have shown health, hygiene, and cosmetic appearance among the most important [7-12]. A study by Williamson and Williamson showed that Midwestern mothers surveyed on their decision to circumcise their sons identified hygiene and appearance as the two major decision-making factors [13]. They found that the circumcision status of the son correlated strongly with the mother’s ideal male partner’s circumcision status for intercourse. Morris et al. expanded on Williamson and Williamson's study by looking into a woman’s preferences for the circumcision of her son across multiple cultures and countries [13,14]. They found that health, disease prevention, and hygiene were cited as the major decision-making factors for circumcision across populations in the United States, Australia, Canada, Sub-Saharan Africa, India, South Korea, China, Japan, and Pacific Islands. A study by the Canadian Pediatric Society reported that women cited health, hygiene, and being like fathers, siblings, or peers to be the main decision-making factors for circumcision [15].

We found that a majority of newborns had fathers that were circumcised, and that the circumcision status of the father is an extremely important factor in the decision-making process. Our study is among the first to provide the specific AAP guidelines policy statement as supplementary educational material to guardians prior to the circumcision procedure as well as surveying factors in deciding to circumcise. The Canadian Pediatric Society found that the percentage of surveyed women stating they received sufficient information about circumcisions ranged from 86.7% to 40.2% [15]. Our study takes this concept one step further by providing guardians with the AAP guidelines and then assessing the usefulness of it as an educational resource.

The AAP’s Task Force on Circumcision recommends clinicians educate guardians on the health benefits and risks of newborn male circumcision in a non-biased way so that guardians can decide if circumcision is in the best interest of their son [5,6]. To that extent, we surveyed guardians on what factors influenced their decision to ultimately pursue circumcision. We learned that in the majority of guardians, the health of their child was the primary deciding factor for circumcision. We sought to learn whether the AAP guidelines policy statement for newborn male circumcision could serve as a resource to supplement the discussion between provider and patient. We showed that not only do most parents read the AAP guidelines, but most find the material helpful for understanding the health benefits of elective circumcision. However, guardians who did not find the AAP guidelines helpful did not change their minds regarding newborn circumcision. This suggests that despite being a useful and informative resource for most parents, the AAP guidelines are most likely not a crucial factor in the decision-making process and therefore shall be used only as an educational resource.

Similar to all survey studies, this study is limited by sampling and response bias. The parents surveyed were from a single geographic area and all sought circumcision prior to participating in the study, which may limit the generalizability of our study. Parent reading and education level were not assessed, limiting the ability to judge whether the parents could comprehend the AAP guidelines. However, the rate of circumcision for our geographic area has been shown to correlate closely to national averages [4]. A study on a national level is needed to confirm our findings and to assess any additional significant factors that may influence which guardians will consider newborn circumcision. Despite our limitations, this study helps to identify some of the more important factors for guardians deciding to circumcise their newborn son and offers insight into an additional resource that may potentially be used as a tool for physicians to counsel guardians regarding the health benefits and risks of newborn circumcision.

## Conclusions

Since male circumcision is such a common procedure in pediatric urology practices, it is vital that clinicians properly educate guardians as well as have resources on hand for guardians to review at a later date. This follows the AAP recommendation to educate guardians on the health benefits and risks of male circumcision. Our results suggest that health is the primary reason why parents decide to circumcise their child, followed by the father of the child being circumcised. Furthermore, we found that most guardians consider the AAP guidelines to be useful. Thus, in addition to education by the provider, providing guardians with this, or similar, information to take home might be beneficial since this allows guardians to make a more informed decision prior to circumcision. Other than maternal age, with younger mothers finding the AAP guidelines useful, there were no significant factors that influence which guardians would consider the AAP guidelines useful or not. Our results suggest that all guardians, especially young mothers, should be provided with information to take home prior to circumcision.

## Appendices

Circumcision Study Survey

1. Are you White, Black or African-American, American Indian or Alaskan Native, Asian, Native Hawaiian or other Pacific islander, or some other race?

- White

- Black or African-American

- American Indian or Alaskan Native

- Asian

- Native Hawaiian or other Pacific Islander

- From multiple races

- Some other race (please specify)

2. Age of the mother?

- Under 18

- 18-24

- 25-34

- 35-44

- 45-54

- 55-64

- 65+

3. Age of the father?

- Under 18

- 18-24

- 25-34

- 35-44

- 45-54

- 55-64

- 65+

4. Did you read the AAP guidelines?

- Yes

- No

5. How helpful was the information from the AAP presented to you in the process of making an informed decision?

- Extremely helpful

- Very helpful

- Somewhat helpful

- Not so helpful

- Not at all helpful

6. Is the father of the child circumcised?

- Yes

- No

7. If applicable, is the brother circumcised?

- Yes

- No

8. What is the main reason why you decided to get your child circumcised?

- Religion (0 = not at all important, 1 = not so important, 2 = somewhat important, 3 = very important, 4 = extremely important)

- Cosmetics (0 = not at all important, 1 = not so important, 2 = somewhat important, 3 = very important, 4 = extremely important)

- Health (0 = not at all important, 1 = not so important, 2 = somewhat important, 3 = very important, 4 = extremely important)

- Other people I know are circumcised (0 = not at all important, 1 = not so important, 2 = somewhat important, 3 = very important, 4 = extremely important)

- Father of the child is circumcised (0 = not at all important, 1 = not so important, 2 = somewhat important, 3 = very important, 4 = extremely important)

- Other (please specify)

## References

[1] Simpson M (2020). Urologic conditions in infants and children: circumcision. FP Essent.

[2] Kogan BA, Feustel PJ (2011). What can we learn from pediatric urology certification logs?. Urology.

[3] Introcaso CE, Xu F, Kilmarx PH, Zaidi A, Markowitz LE (2013). Prevalence of circumcision among men and boys aged 14 to 59 years in the United States, National Health and Nutrition Examination Surveys 2005-2010. Sex Transm Dis.

[4] Owings M, Uddin S, Williams S (2013). Trends in circumcision for male newborns in U.S. hospitals: 1979-2010. NCHS health notes.

[5] American Academy of Pediatrics Task Force on Circumcision (2012). Male circumcision. Pediatrics.

[6] American Academy of Pediatrics Task Force on Circumcision (2012). Circumcision policy statement. Pediatrics.

[7] Ahaghotu C, Okafor H, Igiehon E, Gray E (2009). Psychosocial factors influence parental decision for circumcision in pediatric males of African American descent. J Natl Med Assoc.

[8] Guo YT, Xu B, Chen M (2017). Chinese parents' attitudes toward and their satisfaction with circumcision for 6-14 years old children. (Article in Chinese). Zhonghua Nan Ke Xue.

[9] Leung MW, Tang PM, Chao NS, Liu KK (2012). Hong Kong Chinese parents' attitudes towards circumcision. Hong Kong Med J.

[10] Özveren B (2016). Defining the pathways of parental decision-making and satisfaction levels about newborn circumcision in a setting where traditional male circumcision is prevalent: an online survey study. Urology.

[11] Rediger C, Muller AJ (2013). Parents' rationale for male circumcision. Can Fam Physician.

[12] Spense J, Meller J, Abbey J, Foster K, Sirri C, Naqvi M (2017). Why are we cutting? A survey of cultural views on circumcision in the Texas Panhandle. Glob Pediatr Health.

[13] Williamson ML, Williamson PS (1988). Women's preferences for penile circumcision in sexual partners. J Sex Educ Ther.

[14] Morris BJ, Hankins CA, Lumbers ER, Mindel A, Klausner JD, Krieger JN, Cox G (2019). Sex and male circumcision: women's preferences across different cultures and countries: a systematic review. Sex Med.

[15] Bartholomew S, Boscoe M, Chalmers B (2009). What mothers say: the Canadian Maternity Experiences Survey. Public Health Agency of Canada.

