# Correction: Neonatal Circumcision: What Are the Factors Affecting Parental Decision?

**DOI:** 10.7759/cureus.c137

**Published:** 2023-10-13

**Authors:** Christian G Guevara, Justin K Achua, Ruben Blachman-Braun, Isabella Cabrera-Valencia, George A Ransford, Rafael Gosalbez, Andrew S Labbie, Miguel A Castellan, Alireza Alam

**Affiliations:** 1 Pediatric Urology, Nicklaus Children's Hospital, Miami, USA; 2 Urology, University of Miami Miller School of Medicine, Miami, USA; 3 Urology, Jackson Memorial Hospital, University of Miami Miller School of Medicine, Miami, USA

This article has been corrected to clarify details regarding expiration date of the AAP guidelines distributed to parents as part of this study. The following paragraph has been added to the Materials and Methods section:

It is important to note that the study conducted from January 2020 to April 2020 involved the distribution of the American Academy of Pediatrics (AAP) guidelines as part of the educational process for parents seeking newborn circumcision. However, it should be highlighted that the AAP guidelines available during this study were already expired at the time of its execution. Patients participating in the study were promptly informed that the guidelines were no longer current at the time of the study's execution. To facilitate access to the most up-to-date and relevant information, patients were directed to visit the official AAP website. Here, they could readily find the latest guidelines, recommendations, and comprehensive information, thereby enabling them to make informed decisions based on the most current data available.

